# Indirect and atypical imaging signals of endometriosis: A wide range of manifestations

**DOI:** 10.52054/FVVO.13.4.048

**Published:** 2021-12-30

**Authors:** A Vigueras Smith, R Cabrera, C Trippia, M Tessman Zomer, W Kondo, H Ferreira, L Carttaxo Da Silva, R Sumak

**Affiliations:** Department of Gynaecology, Vita Batel Hospital, Curitiba, Brazil; Department of Radiology, Nossa Senhora das Graças Hospital. Curitiba. Brazil; Department of Gynaecology, Centro Hospitalar Universitario Do Porto, Universidade do. Minho, Porto Portugal; Department of Radiology, Hospital São Luiz Itaim, São Paulo, Brasil

**Keywords:** Endometriosis, imaging, ultrasound, diagnosis

## Abstract

**Background:**

Whilst some imaging signs of endometriosis are common and widely accepted as ‘typical’, a range of ‘subtle’ signs could be present in imaging studies, presenting an opportunity to the radiologist and the surgeon to aid the diagnosis and facilitate preoperative surgical planning.

**Objective:**

To summarise and analyse the current information related to indirect and atypical signs of endometriosis by ultrasound (US) and magnetic resonance imaging (MRI).

**Methods:**

Through the use of PubMed and Google scholar, we conducted a comprehensive review of available articles related to the diagnosis of indirect signs in transvaginal US and MRI. All abstracts were assessed and the studies were finally selected by two authors.

**Results:**

Transvaginal US is a real time dynamic exploration, that can reach a sensitivity of 79-94% and specificity of 94%. It allows evaluation of normal sliding between structures in different compartments, searching for adhesions or fibrosis.

MRI is an excellent tool that can reach a sensitivity of 94% and specificity of 77% and allows visualisation of the uterus, bowel loop deviation and peritoneal inclusion cysts. It also allows the categorisation and classification of ovarian cysts, rectovaginal and vesicovaginal septum obliteration, and small bowel endometriotic implants.

**Conclusion:**

The use of an adequate mapping protocol with systematic evaluation and the reporting of direct and indirect signs of endometriosis is crucial for detailed and safe surgical planning.

## Introduction , epidemiology and clinical features

Endometriosis is estimated to affect approximately 10% of the female population and its prevalence in patients with chronic pelvic pain can reach up to 82% (Kondo et al., 2011). The final diagnosis is histological and only pelvic surgery with concurrent biopsy can confirm it. The lack of non-invasive diagnostic methods has limited our knowledge about the natural history and real epidemiology and can hinder the physician in choosing the optimal treatment. Hence, studies on prevalence and risk factors have been based mostly on clinically diagnosed cases, biased by missing a significant number of patients and therefore limiting their validity (Eskenazi and Warner, 1997).

One of the major problems in the management of endometriosis is the delay in diagnosis, and often symptoms can occur for 6 to 10 years before an adequate diagnosis is made (Fauconnier et al., 2013). The non-invasive methods available for the diagnosis of endometriosis include transvaginal ultrasound (TVUS), magnetic resonance imaging (MRI), transrectal ultrasound and serum quantifications of biomarkers such as angiogenesis/ growth factors, apoptosis markers, cellular adhesion molecules, hormonal markers, immunity/ inflammatory markers, oxidative stress markers, micro RNAs, tumour markers (CA-125) and other proteins (Nisenblat et al., 2016). Biomarkers have different diagnostic values, but they all appear to be suboptimal (Olive et al., 1993).

Imaging modalities used for the preoperative diagnosis are key to mapping the anatomical extension of the disease and allowing adequate presurgical planning and appropriate patient counselling. These exams could be used alone or combined, depending on the hospital or surgical team protocols (Fraser et al., 2015; Sayasneh et al., 2015)

According to the Cochrane review by Nisenblat et al. (2016), that included over 4800 women, the sensitivity and specificity of TVUS was 93%-96% and 79%-94% for endometriomas and deep endometriosis (DE) lesions respectively. Meanwhile MRI had a global sensitivity and specificity of 94% and 77% respectively for DE lesions. The selection of which imaging modality to use must consider the clinical scenario and the advantages/disadvantages of each imaging technique (Table I).

**Table I:** TVUS and MRI comparison (Dumontier et al., 2000; Gonçalves et al., 2021; Bazot et al., 2011).

Imaging exam	Advantages	Disadvantages
Transvaginal ultrasound	Low-cost	Requires training and expertise
	Dynamic aspect of ultrasound examination	Challenging retrospective review of images
	Better detection of Pouch of Douglas lesions	Difficulty in diagnosing small endometriomas
	Better in identifying intestinal lesions	More discomfort for patients during examination
Magnetic resonance imaging	Mapping of disease outside of pelvis	Limited assessment of the right iliac fossa
	Better detection of uterosacral ligament lesions	Static images, it is not possible to mobilisze the pelvic structures
	Retrospective review of images when second opinion is needed	High cost
	Virgin patients	Limited evaluation of small intestinal lesions
	Best for evaluation of the pelvic floor and innervation	

It is accepted that the success of the surgical procedure is directly related to the complete excision of endometriotic lesions, therefore precise information prior to surgery, regarding the number, size, and anatomical distribution of all endometriotic implants, along with the depth of infiltration, degree of bowel lumen stenosis and distance from anal verge in cases of bowel involvement is absolutely necessary when planning surgical treatment (Practice Committee of the American Society for Reproductive Medicine, 2014).

The purpose of this review is to present and analyse the current information related to ultrasound and magnetic resonance findings in endometriosis patients. We aim to review the wider literature and describe practice in high volume endometriosis centres.

## Material and methods

A comprehensive review of the literature was carried out for English publications in Pubmed and Google Scholar from 1992 - 2021, related to the diagnosis of deep endometriosis using indirect signs in transvaginal ultrasound and magnetic resonance.

We finally included 68 studies found under the search of following MeSH and the key- words terms: Endometriosis AND Imaging OR Ultrasonography OR Magnetic resonance imaging OR Diagnostic imaging.

All abstracts were reviewed, and the studies were finally selected by two authors (AV. and RC.) according to the aim of this review.

Initially, a structured investigation question was created using the PICO strategy as shown in Table II.

**Table II:** Investigation structure of this review.

P.I.C.O. Structured Investigation Question	
**P** (Patient/Problem)	Patients undergoing imaging studies for deep endometriosis diagnosis
**I** (Intervention)	Mapping for deep endometriosis by ultrasound (abdominal and transvaginal) with/without bowel preparation, MRI (pelvic and abdominal) with/without bowel preparation for diagnosis of deep endometriosis lesions.
**C** (Comparison)	No comparison
**O** (Outcome)	Diagnostic accuracy of MRI and ultrasound, describe the main indirect and atypical signals of endometriosis for each pelvic compartment and pelvic adhesions

## Describing an optimal ultrasound mapping for endometriosis lesions

Transvaginal ultrasound is the first line imaging study when endometriosis is suspected. This exam allows the visualisation of DE lesions in almost all anatomic sites and compartments, but its accuracy is dependent on the operators’ knowledge and skills, along with the quality of ultrasound equipment. Potential disadvantages are the reduction of the diagnostic accuracy for posterior lesions above the rectosigmoid junction and the severe discomfort and pain that can reach up to 25% of patients examined (Schiffmann et al., 2014).

Ultrasound examination must evaluate all implants in both the anterior and postero–lateral anatomical compartments, according to the classification reported by Chapron et al. (2003). A good mapping should explore at least the bladder, rectovaginal septum, uterosacral ligaments, torus uterinus, posterior vaginal fornix, rectum, rectosigmoid junction, parametria and ureter (Exacoustos et al., 2014). Some basic actions must be taken in consideration. For bladder exploration, a moderately full bladder is required for optimal visualisation. The distal ureter must be identified at the pelvic brim, crossing the common iliac arteries and adjacent to the bladder trigon lateral to the cervix.

Pre-exam standard protocol usually includes a low residue diet the day before, a 4 hour fasting before the study and intestinal preparation using 130 ml of phosphate enema, two 70%-sorbitol suppositories and laurisulphate of sodium one hour prior to the examination. For better evaluation of the rectovaginal septum and vaginal vault, 60 ml of intravaginal ultrasound gel can be applied.

In our unit, ultrasound is performed both transvaginally and transabdominally, following a standard operative protocol to avoid bias. The importance of the abdominal ultrasound resides not only in the diagnosis of extrapelvic endometriosis within the right iliac fossa (appendix - caecum - right bowel) which might be as high as 3% (Gustofson et al., 2008), but also with regards to diaphragmatic implants (0.1-1.5%), especially in cases where severe pelvic disease is present (Vigueras et al., 2020). The diagnosis of extra pelvic disease can be extremely challenging and may result in further delays and incomplete surgical treatment (Andres et al., 2019).

A systematic evaluation of three compartments and twelve areas is undertaken: the anterior compartment (bladder and distal portion of ureter, anterior uterine serosa, round ligaments, vesicovaginal and vesico-uterine septum), medium compartment (adnexal) and posterior compartment (retrocervical area and pouch of douglas, posterior vaginal fornix, uterosacral ligaments, rectosigmoid colon, rectovaginal septum). Abdominal ultrasound evaluates the upper portion of sigmoid, caecum, terminal ileum, appendix, kidneys, diaphragm and abdominal wall. Among patients with intestinal involvement, Dousset et al. (2010) highlighted up to 24 % of patients will have DE lesions involving the right iliac fossa (appendix, caecum, ileum).

For intestinal implants, we report size (longitudinal and transverse measurements), number of lesions (presence of multifocal or multicentric disease), depth of penetration, involved circumference and distance from anal verge. Recently, Di Giovanni et al. (2018) highlighted a 100% sensitivity and 91% specificity of the combined transvaginal and abdominal ultrasound in the diagnosis of DE bowel nodules, supporting the use of the combined approach.

A meta-analysis undertaken by Guerriero et al. (2015), showed that the detection of endometriosis in the uterosacral ligament, had a pooled sensitivity and specificity of 53% (95%CI, 35–70%) and 93% (95%CI, 83–97%), respectively. For detection of endometriosis in the rectovaginal septum, the overall pooled sensitivity and specificity were 49% (95%CI, 36–62%) and 98% (95%CI, 95–99%), respectively. For detection of vaginal endometriosis, the overall pooled sensitivity and specificity were 58% (95%CI, 40–74%) and 96% (95%CI, 87–99%), respectively. For detection of bladder endometriosis, the overall pooled sensitivity and specificity were 62% (95%CI, 40– 80%) and 100% (95%CI, 97–100%), respectively.

Direct assessment of the indirect signals of adhesions and atypical manifestations is performed, searching for inclusion peritoneal cysts, hyperechogenic ovarian wall foci with associated thickening of peritoneal layer at the ovarian fossa, a fixed ante- or retroverted uterus, medial and/or posterior retraction of the ovaries, fixation of the ovaries to the ipsilateral ovarian fossa, retraction of the bowel toward the DE lesion, elevation of vaginal fornix and absence of sliding signs.

## Describing an optimal MRI mapping for endometriosis lesions

MRI is recognised as a valuable tool for diagnosis, presurgical planning, and detection of extra pelvic disease, hence determining whether the patient will require multidisciplinary treatment (Andres et al., 2019). It is particularly useful in cases where obliteration of anterior and posterior peritoneal spaces limits direct visualisation by laparoscopy, a scenario that could change diagnosis and management (Jha et al., 2019; Moro et al., 2019). Compared to TVUS, image acquisition is more reproducible and images acquired encompass a larger field of view, allowing detection of disease outside the pelvis. Additionally, information gathered from multiple sequences allows more specific characterisation of ovarian lesions (Jha et al., 2019).

Our standard MRI protocol was previously presented in the literature, and can be summarised as follows (Fiaschetti et al., 2018; Kondo et al., 2011).

- Pre-exam preparation is quite similar to the TVUS protocol, the use of 60 ml of ultrasound gel in the vagina for better visualisation of the vaginal fornices and the posterior compartment spaces and so bowel preparation with phosfoenema 130 ml is given one hour before the exam. Also, an intravenous injection of a combination of hyoscine- N-butylbromide and sodium dipyrone (Buscopan MR) to reduce bowel movements and uterine contractions is administrated immediately before examination (Dunselmann et al., 2014; Manganaro et al., 2012).
- Precontrast images include the acquisition of axial, sagittal and coronal T2-W fast spin echo images and axial T1-W gradient echo images in and out of phase and with fat suppression.
- Post contrast images are obtained following an intravenous administration of a paramagnetic contrast agent gadolinium chelate (Dotarem) at a dose of 0.2 mmol/kg and followed by the acquisition of axial and sagittal volumetric fatsaturated T1-W sequences.

Other MRI techniques such as, tractography, DTI and DWI sequences could be used as a part of the mapping. Tractography allows us to study the threedimensional architecture of nerve fibres. It is able to reconstruct nerve fibres starting from diffusion-weighted images. In endometriosis, diffusion techniques and tractography are useful as a non-invasive assessment of pelvic nerves and their pathway, that might be involved by posterior DE nodules. (Yang et al., 2011; Manganaro et al., 2014). Presence of pathological DTI tractography findings of the sacral nerve roots correlates with the type of pain, adhesions and DE (Porpora et al., 2018; Zhang et al., 2017).

With T3 MRI system, without the use of any patient preparation such as laxatives, anti- spasmodics, vaginal or rectal contrast distention, identification of rectosigmoid DE shows sensitivity of 94% and specificity 95% (Yap et al., 2018).

## Direct and indirect imaging features of endometriotic lesions

In general, it is accepted that endometriosis has three clinical presentations: peritoneal endometriosis, ovarian endometrioma and DE lesions (Nisolle and Donnez, 1997).

Direct features of pelvic endometriosis are the endometrioma and the DE nodular / plaque like lesions. Indirect features consist of a wide range of manifestations (Table III). Classically, these DE lesions can be presented in two major morphological patterns, in both MRI and TVUS (Kondo et al., 2011).

**Table III:** Main indirect imaging signals of pelvic endometriosis.

Rectum: Tethered appearance	Kissing ovaries	Ovarian fossa thickening
Periovarian collections	Uterine deviation	Medalisation of round ligaments
Focal bowel skip	Bowel loop angulation	Loss of fat planes between structures
Asymmetrical thickness of uterosacral ligaments	Peritoneal inclusion cysts	Hyperechogenic ovarian cyst wall foci
Haematosalpinx - Sactosalpinx- Tubo-ovarian complex	Elevation of posterior fornix	Ovarian fluid - fluid levels

1. **Nodular Lesion:** Retractile or Nonretractile / Regular or irregular margins / With or without endometrial glands.
2. **Plaque- Like Lesion:** Retractile, Infiltrative and with nondefined margins.

## Indirect and atypical signals according to anatomical compartments

### Ovarian endometrioma

**Figure 1:**
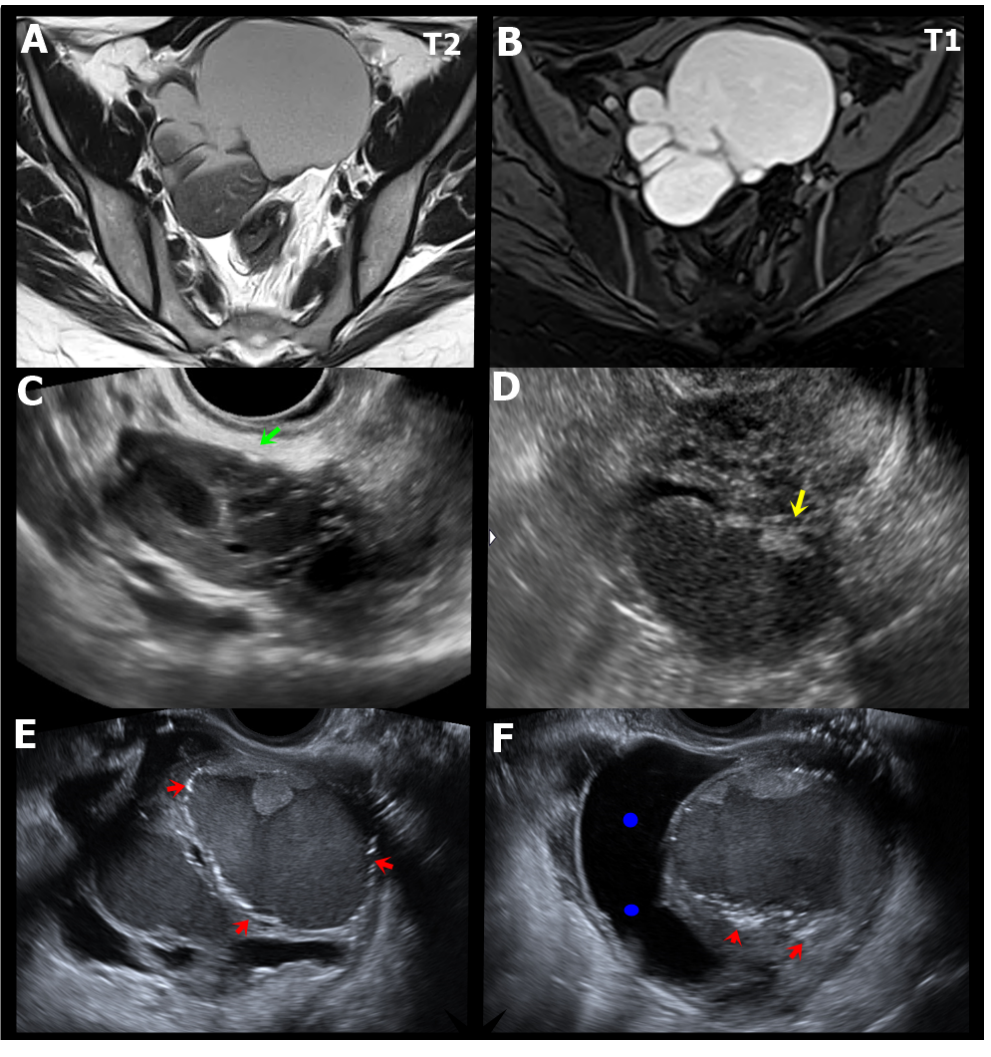
MRI and ultrasound appearances of ovarian endometriomas. A. Axial T2W MR image shows a multiloculated endometrioma with variable appearances of “T2 shading”. B. Axial T1W MR image with fat saturation shows a multiloculated endometrioma with marked and uniform hyperintensity with a “light bulb bright” appearance. C. Transvaginal ultrasound (TVUS) shows a small endometrioma with small echogenic focus in the ovarian parenchyma (green arrow).D. TVUS) shows an ovarian endometrioma with heterogeneous content due to small papillary projection (yellow arrow). E. TVUS shows two ovarian endometriomas, the largest with irregular contours and hyperechogenic focus in the wall (red arrows).F. TVUS showing an ovarian endometrioma with irregular contours and hyperechogenic focus in the wall (red arrows) associated with paraovarian inclusion cyst seen as an indirect sign of adherence process (blue dots).

The classic TVUS finding is described as an unilocular or multilocular (less than five) cyst with the classical “ground glass” homogeneous low-level echogenicity within the cyst, and peripheral flux on the doppler exploration (Figure 1). (Guerriero et al., 1998). Nevertheless, almost 50% could present other “atypical” ultrasound characteristics.

The hyperechogenic/ hypointense cyst wall foci is seen in about 35% of cases. For some authors, the origin is cholesterol sediments similar to those found in gallbladder wall, while for others it is just hemosiderin deposits or calcification. (Figure 1) (Van Holsbeke et al., 2010; Teixeira et al., 2013). This sign is seen exclusively on the cyst wall, and is a fundamental clue for the right diagnosis (Testa et al., 2011; Reid and Condous, 2017).

Finally, the “fluid – fluid” levels suggest a recent haemorrhagic event, and a complementary post menstrual TVUS is recommended to ensure the correct diagnosis is reached.

The main MRI signal is called the “shading sign” (Figure 1), a high signal on T1-W and variable low signal on T2-W images, caused by the elevated iron and protein content within the lesion due cyclical bleeding, varying from a complete to a weak sign (Siegelman and Oliver, 2012) Likewise, this lesion can demonstrate restricted diffusion and low ADC values due the “T2 blackout effect” usually seen. Besides this classic presentation, indirect signals could appear requiring exhaustive imaging exploration for a correct diagnosis (Table IV).

**Table IV:** Indirect signals of ovarian endometriosis.

Technique	Signals	
Transvaginal Ultrasound	Hyperechogenic cyst wall foci without acoustics shadow	Fluid - fluid levels
	Thickening of cyst wall and peritoneal layer of ovarian fossa	
Magnetic Resonance Imaging	Hypointense cyst wall foci in T2-W Complex echotexture	Peri-ovarian inclusion cysts seen as irregular hyperintense formation on T2-W

**Figure 2:**
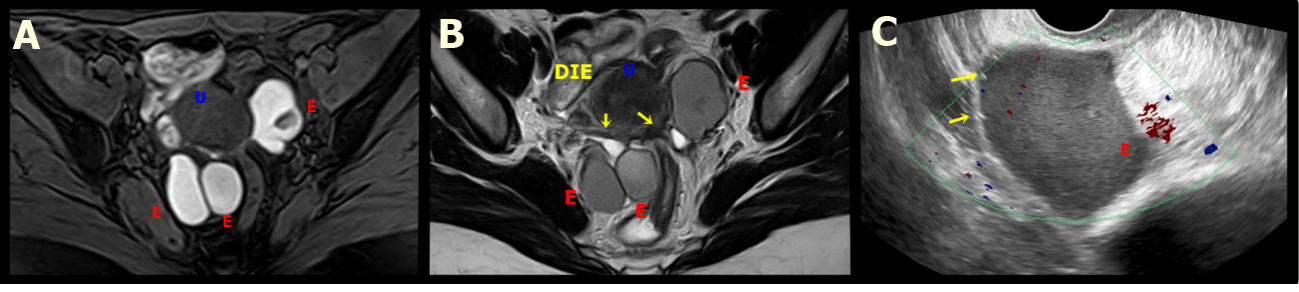
A. Axial T1W MR image with fat saturation shows bilateral ovarian endometriomas with marked and uniform hyperintensity with a “light bulb bright” appearance, demonstrating hematic content. U: Uterus. B. Axial T2W MR image shows bilateral ovarian endometriomas (E) with decreased signal on T2W (T2 shading) that reflects the varying chronicity of blood products from repeated haemorrhage. Yellow arrows show a deep endometriosis plaque in the retrocervical space, with extension to the left ovarian fossa and anterior rectal wall. U: UterusC. Transvaginal ultrasound showing a unilocular cyst with regular walls and homogeneous ground glass echogenicity, compatible with endometrioma (E) with small hyperechogenic focus in the wall (yellow arrow).

The complex echotexture may include solid components (blood clots) and thick septums (Figure 2). Decidualised endometrioma is a reaction that occurs during pregnancy. Approximately 10-12% of endometriomas undergo decidualisation. This is caused by the ectopic endometrium within an endometrioma that undergoes the same transformation as the endometrium within the uterus into a specialised type of endometrium designed to support pregnancy (Craig et al., 2019).

### Posterior compartment endometriosis

According to Hudelist et al (2011), bowel wall has four layers: serosa, smooth muscle layer, submucosa and mucosa. The first two appear as thin hypoechogenic lines, while mucosa is visualised as a hyperechogenic contour covering the rectal smooth muscle.

**Figure 3:**
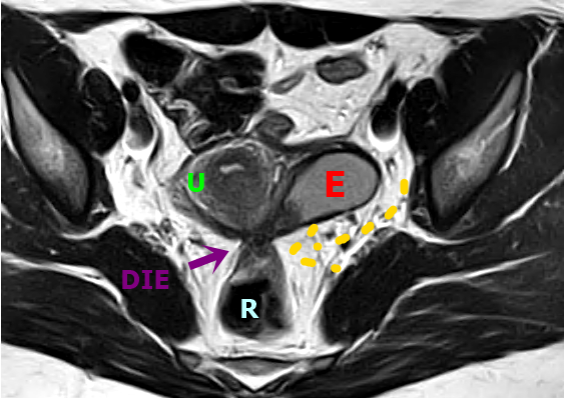
Axial T2W MR image shows a deep endometriosis (DE) plaque in the posterior uterus with adhesions that extend from the torus uterus, invading the wall of the rectum and promoting retraction and medialisation of the left ovary that contains endometrioma (E). Bowel-invasive endometriosis of the rectum is also present with a “mushroom cap” lesion. U: Uterus, E: Endometrioma, R: Rectum, DIE: DE plaque.

**Figure 4:**
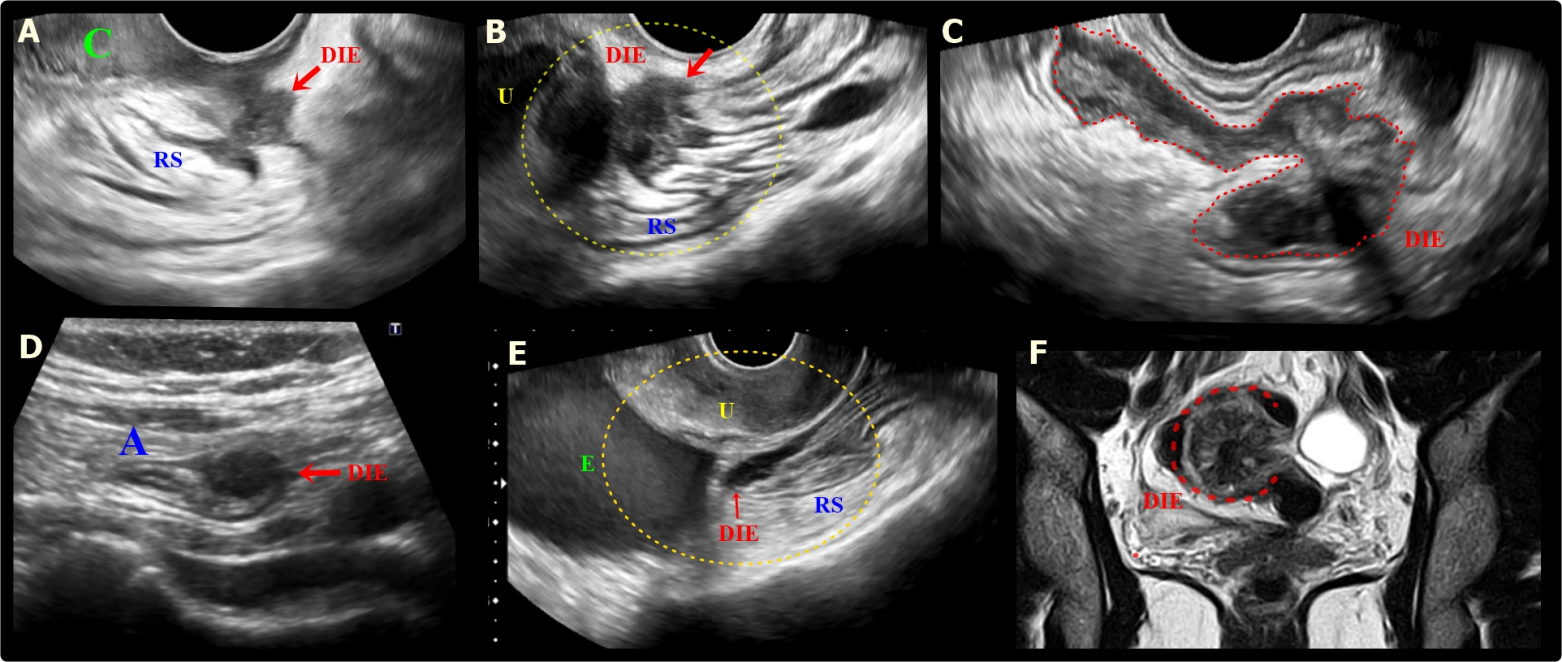
A. Sagittal transvaginal ultrasound (TVUS) image shows a hypoechoic endometriotic nodular lesion, with irregular margins, infiltrating the low retrocervical space, with extension to the posterior vaginal fornix and the serosa of the anterior rectal wall (RS) (red arrow). DIE: deep endometriosis. B. Sagittal oblique TVUS image shows a hypoechoic lesion (red arrow) with ill-defined margins, covering and infiltrating the posterior uterine wall and the anterior rectosigmoid wall (RS), causing retraction and angulation of this segment. U: Uterus. C. Sagittal oblique TVUS image shows a large hypoechoic endometriotic lesion in plaque (red dashed) infiltrating the anterior wall of the rectosigmoid colon. D. TVUS image demonstrates a hypoechoic nodule (red arrow) infiltrating the appendiceal tip (A). E. Sagittal oblique image of TVUS shows a hypoechoic endometriotic lesion infiltrating the retro- and paracervical space, with extension to the anterior wall of the rectosigmoid colon (RS) (red arrow). Also note, the presence of an ovarian endometrioma (E). U: Uterus. F. Coronal T2W MR image shows deep endometriosis lesion with archiform morphology in the rectosigmoid (red dashed).

Despite the typical appearance of intestinal DE nodule as a solid hypoechoic retractile lesion penetrating and distorting the normal anatomy (TVUS), and the classic heterogeneous “low signal” intensity of the hypertrophic muscularis propria, covered with a “high signal” intensity in the submucosa known as the “mushroom” image on T2-W MRI (Figure 3), indirect signals of intestinal impairment could also be observed (Table V, Figure 4). The main dynamic sign in TVUS is the obliteration of posterior cul-de-sac, assessed by the absence of the “sliding sign” of anterior rectum over the posterior cervix.

**Table V:** Indirect signals of intestinal endometriosis.

Technique	Signals	
Transvaginal Ultrasound	Bowel wall thickening with anterior triangular retraction of rectum toward torus uteri	Dynamic real time evaluation
Magnetic Resonance Imaging	Focal hyper-intense bowel wall foci in T1 and T1-W	

**Figure 5:**
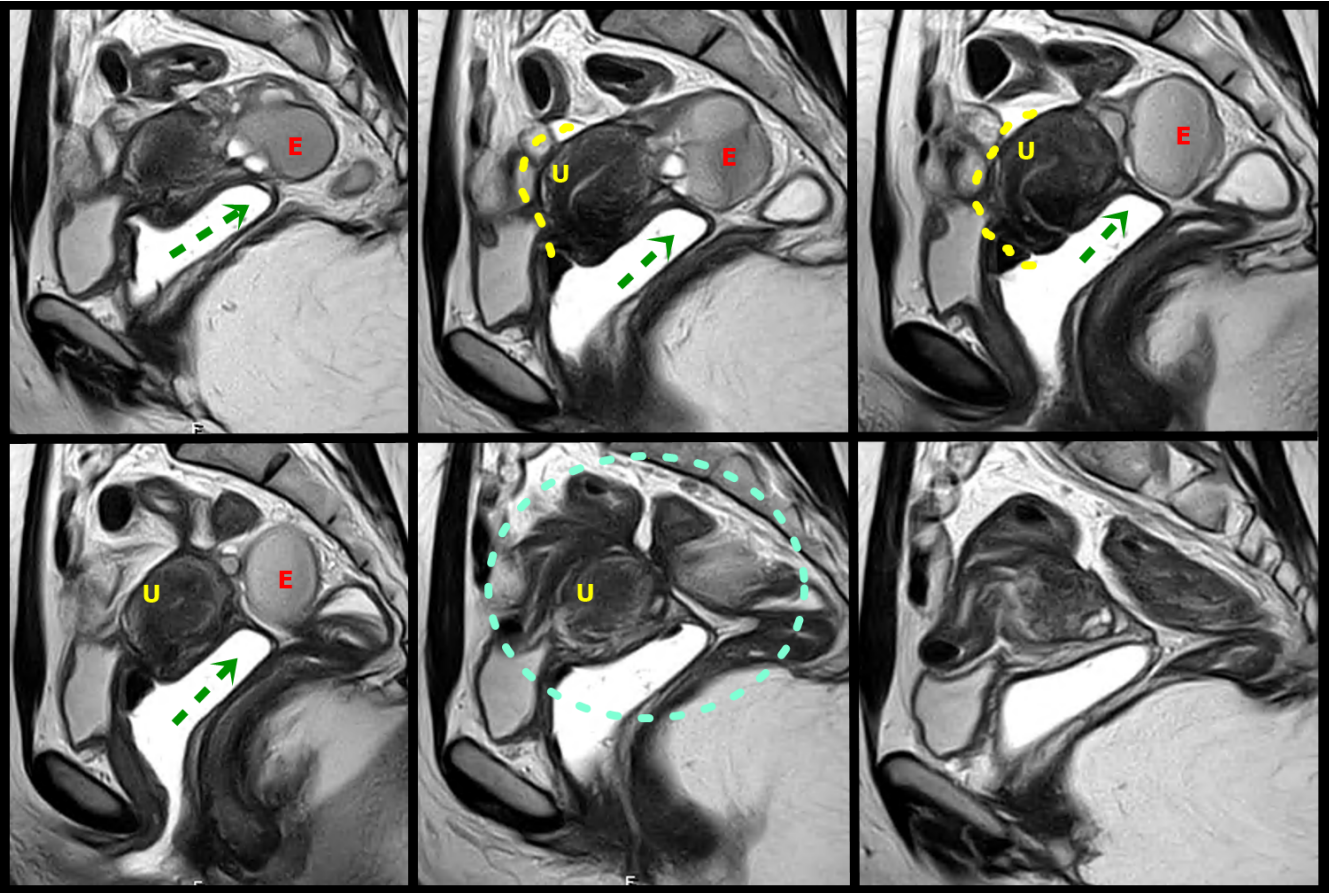
Sagital T2W MR images with distension of the vagina by aqueous gel show a plaque in the rectouterine and retrocervical space, invading and elevating of the posterior vaginal fornix (green arrow), causing obliteration of the posterior cul-de-sac (green circle). The lesion infiltrated the posterior uterine serosa and myometrium and is causing uterine retractile retroflexion (yellow interrupted line)A left adnexal endometrioma (E) demonstrates “T2 shading” and associated fibrotic change. U uterus.

**Figure 6:**
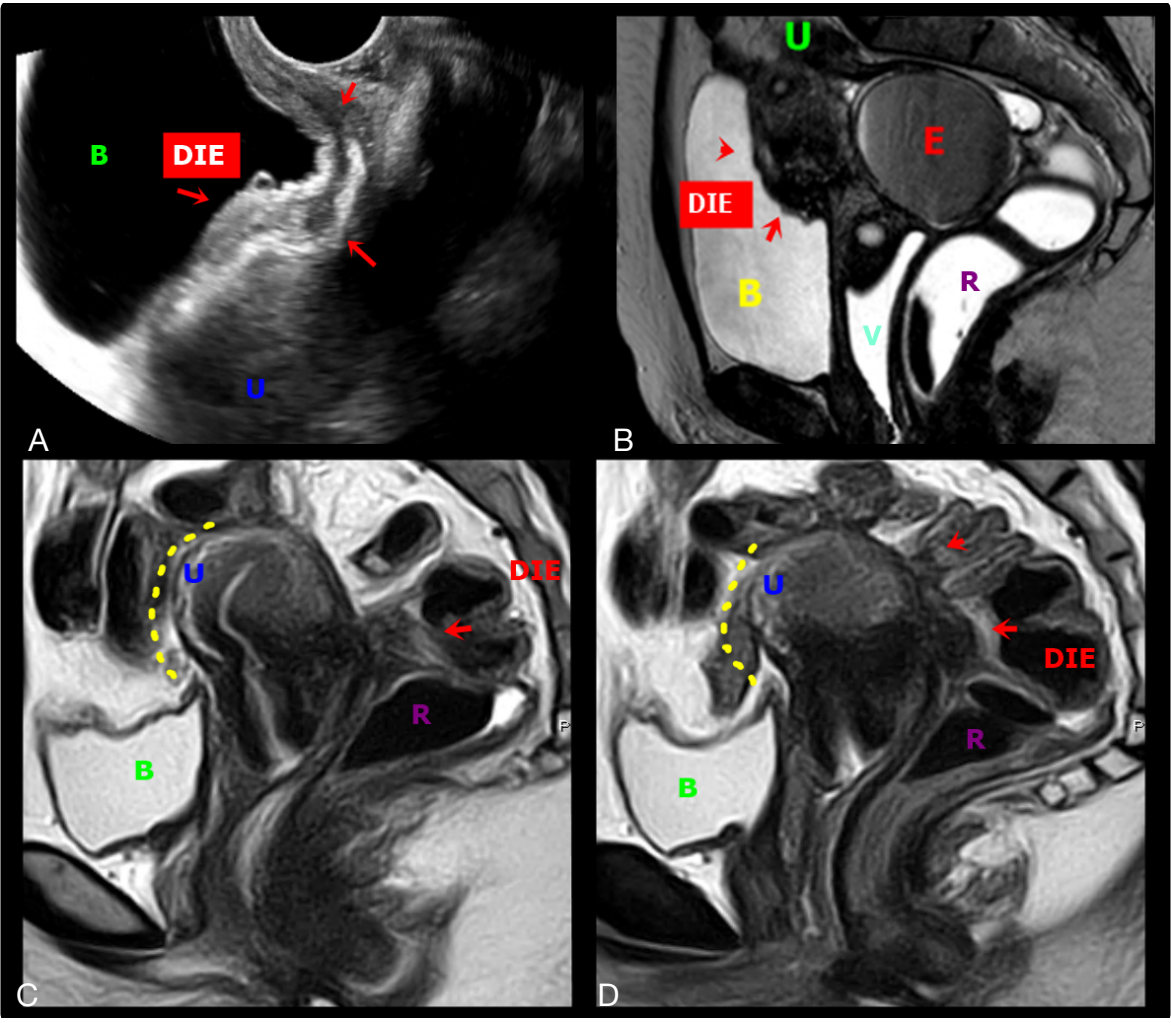
A & B. Transvaginal ultrasound and axial T2-weighted MR images show deep endometriosis (DIE, red arrows) obliteration of the peritoneum of the vesico-uterine space with involvement of the posterosuperior bladder wall. The lesion infiltrates the peritoneum of the vesicouterine space, causing the obliteration of the fatty planes between adjacent structures. B: Bladder, U: Uterus, R: Rectum, E: Endometrioma, V: Vagina; C & D. Sagittal T2W MR images show a deep endometriosis (DIE) plaque in the retrocervical space with bowel involvement, causing obliteration of the posterior cul-de-sac with loss of fat planes between adjacent structures and forced retroflexion of the uterine fundus (yellow interrupted line). B: Bladder, U: Uterus, R: Rectum, E: Endometrioma

Cul-de-sac involvement is present in almost 69% of patients with endometriosis (Foti et al., 2018). In this scenario, the role of the MRI is important since TVUS can miss up to 40% of the lesions (Figure 5,6) (Macario et al., 2012).

The ability to diagnose rectal/rectosigmoid DE and pouch of Douglas obliteration with TVUS is dependent on operator training, skills and experience (Reid and Condous, 2017). Even when a DE implant is diagnosed, the findings of this will be observed as an ‘indirect imaging sign’, since this represents the response to a primary lesion located in the anatomical place (Table VI).

**Table VI:** Indirect signals of pouch of Douglas (cul de sac) endometriosis.

Signs	Characteristics	
Loss of Tissues Interface	Disappearance of the fat tissue that separates anatomical structures (MRI) Hypointense thickening bands with distortion of surrounding organs on T2-W (MRI)	
Thickening Bands Appearance	Retroflexed uterus (US - MRI)	Rectum tethered appearance (MRI)
Obliteration Signals	Elevation of posterior fornix (US - MRI) Strands between uterus and bowel (US - MRI)	Fibrotic plaque covering uterine serosa (US - MRI) Asymmetrical thickness of USL giving an archiform and tether appearance (US - MRI )

### Anterior compartment endometriosis

Bladder focal lesions are diagnosed as round shaped lesions, with or without cystic are-as protruding towards the bladder lumen, mostly in the posterior bladder wall and closer to vesico-vaginal pouch. MRI usually shows an irregular hypo-intense image in T2-W at sagittal plane (Table VII, Figure 6)

**Table VII:** Indirect signals of bladder endometriosis.

Technique	Signals	
Transvaginal Ultrasound	Vesicouterine pouch obliteration (US - MRI)	Dynamic real time evaluation (US)
	Medialisation of round ligaments (US - MRI)	
Magnetic Resonance Imaging	Tiny hyperintense spots in T2-W inside the lesion, representing dilatations of endometrial glands	Hyper-intense bladder wall foci in T1-W

**Figure 7:**
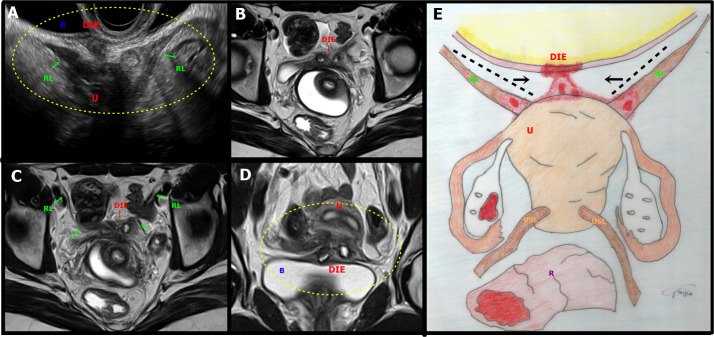
A. Sagittal oblique transvaginal ultrasound image shows a hypoechoic endometriotic lesion with irregular and ill-defined margins that infiltrated and obliterated the peritoneum of the vesicouterine space, extending to the insertion of the round ligaments (RL), more evident on the right. B: bladder, DIE: deep endometriosis, U: uterus, RL: round ligament. B-D. Axial (B, C) and coronal (D) T2-weighted MR images show irregular thickening and medialisation of the round ligaments (low signal intensity) at their insertion sites, near the uterus. Focal thickening of the detrusor muscle of the bladder and the anterior uterine serosa is also seen. Abbreviations as in A. E. Illustrative figure showing a focus of deep endometriosis (DIE) in the anterior compartment of the pelvis. R: Rectum, U: Uterus, RL: Round ligament, USL: Uterosacral ligaments.

The anterior cul-de-sac obliteration is seen when there is an extension of a primary vesico-uterine pouch plaque-like lesion to the bladder (Figure 7).

The main dynamic sign in TVUS is the loss of the “sliding sign” between the bladder and the uterus. Involvement of the round ligament is less common however it requires assessment, particularly when bladder is affected. The typical findings are the medialisation of both ligaments (“V” shape appearance) and the focal or global ligament thickening (higher than 6 mm), appearing as hypo- intense image on T1 and T2-W sequences. (Manganaro et al 2015). Other indirect images are less frequently seen (Table VIII).

**Table VIII:** Indirect signals of round ligament endometriosis

Technique	Signals	
Magnetic Resonance Imaging	Nodular hyper-intense lesion in T1-W Lateral deviation of the uterus in unilateral involvement (US - MRI)
Transvaginal Ultrasound	Thickened “ V shape” image of round ligaments, when bladder and bilateral round ligaments are compromised (US - MRI)

### Ureter

**Figure 8:**
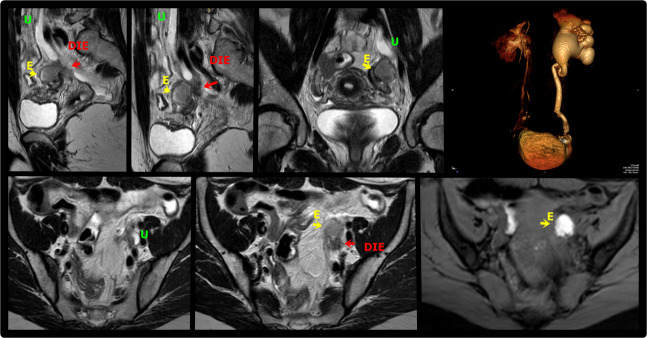
Sagittal (A, B), coronal (C) and axial (E,F) T2W and axial T1W with fat saturation (G) MR images demonstrate deep endometriosis thickening of the left paracervical area(arrow) involving the parametrium and extending to the ovarian fossa and adjacent ureter, resulting in upstream hydroureter and hydronephrosis. There is also an endometrioma in the left ovary (E). MR urogram volumetric reconstruction shows asymmetry of the ureters with accentuated hydronephrosis to the left (D). DIE: deep endometriosis, U: Ureter, E: Endometrioma.

In the case of ureter involvement, the classical tubular anechoic image of the pelvic ureteral dilatation due to extrinsic or intrinsic stenosis, is easy to demonstrate by MRI urography with 2D T2-W sequences or delayed contrast enhanced 3D sequences (Lakhi et al., 2014) (Figure 8). Excluding this, the diagnosis is laborious and must be suspected in all bulky (more or equal to 3 cm diameter) central or lateral and posterior nodules (Donnez et al., 2002). The major indirect sign is when interface of fat between the ureter and the nodule is no longer visible in the T2-W sequence (Takeuchi et al., 2008).

### Fallopian Tube

When the fallopian tubes are affected by endometriosis, in addition to the direct visualisation of a hydrosalpinx with the classic “beads on string” image on cross sectional exploration (Timor-Tritsch et al., 1998), indirect manifestations can also be observed as follows.

1. Sactosalpinx, a dilated tube with thick walls and incomplete septa, fulfilled with dense fluid content (Timor-Tritsch et al., 1998)
2. Haematosalpinx, usually seen as a hyperintense tubal fluid on T1-W.
3. Tubo-ovarian complex, when both the ovary and the fallopian tube are compromised.

### Pelvic adhesions

Peritoneal adhesions are defined as a formation of bands of fibrous tissue between intra-abdominal organs or peritoneal surfaces, resulting from a healing process after traumatic, ischaemic, irritative or infectious pathologies.

**Figure 9:**
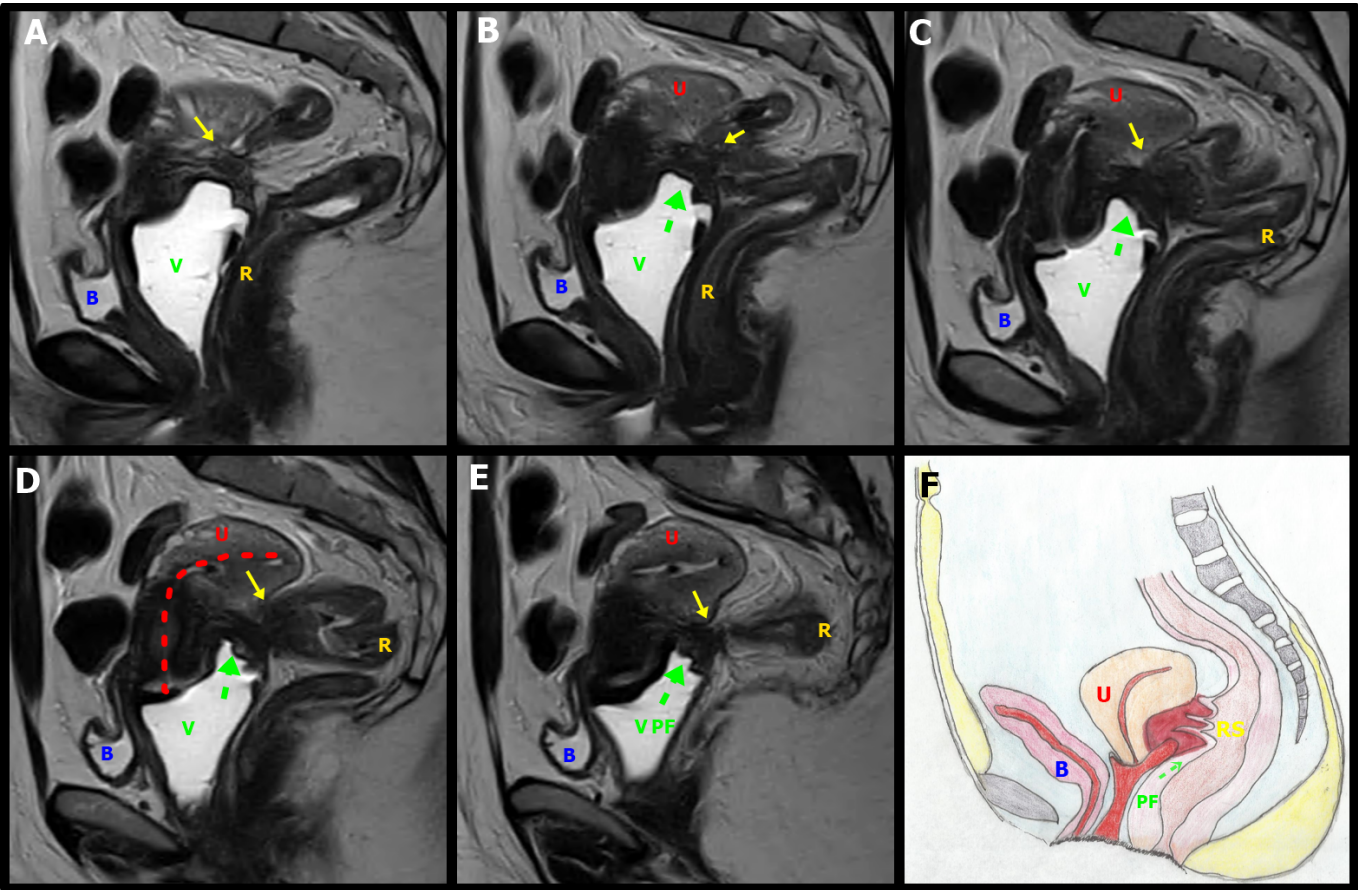
A-E. Sagittal T2W MR images with distension of the vagina (V) by aqueous gel show a stellate low-signal-intensity endometriotic lesion containing small cystic areas in the retrocervical space, with indirect signals of adherence; infiltration of the bilateral uterosacral ligaments, elevation of the posterior vaginal fornix (green arrow),causing obliteration of the posterior cul-de-sac and uterine retractile retroflexion (red interrupted line). Bowel-invasive endometriosis of the rectum is also present (yellow arrows). F. Schematic representation of the imaging signs shown in A-E sequences: Vagina, C: Cervix, U: Uterus, B: Bladder, RS: Rectosigmoid, PF: Posterior Fornix.

**Figure 10:**
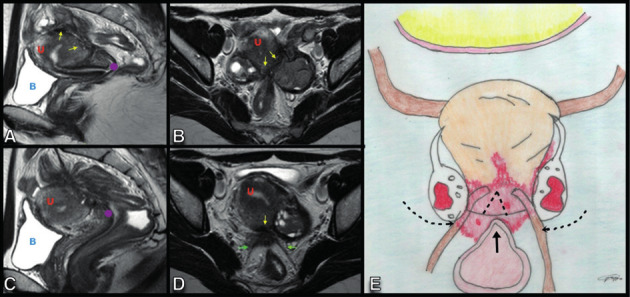
Sagittal (A,C) and axial (B, D) T2W MR images show a deep endometriosis lesion in the retrocervical space, in the topography of the uterine torus (yellow arrow) and insertion of the uterosacral ligaments (green arrow) with extension to the peritoneum of the ovarian fossae with posteriorisation and medialisation of the ovaries (“Kissing ovaries”). There is also extension of the lesion to the rectosigmoid colon with cranial angulation and retraction in the loop segment involved. DIE: Deep Endometriosis, U: Uterus, B: Bladder.

**Figure 11:**
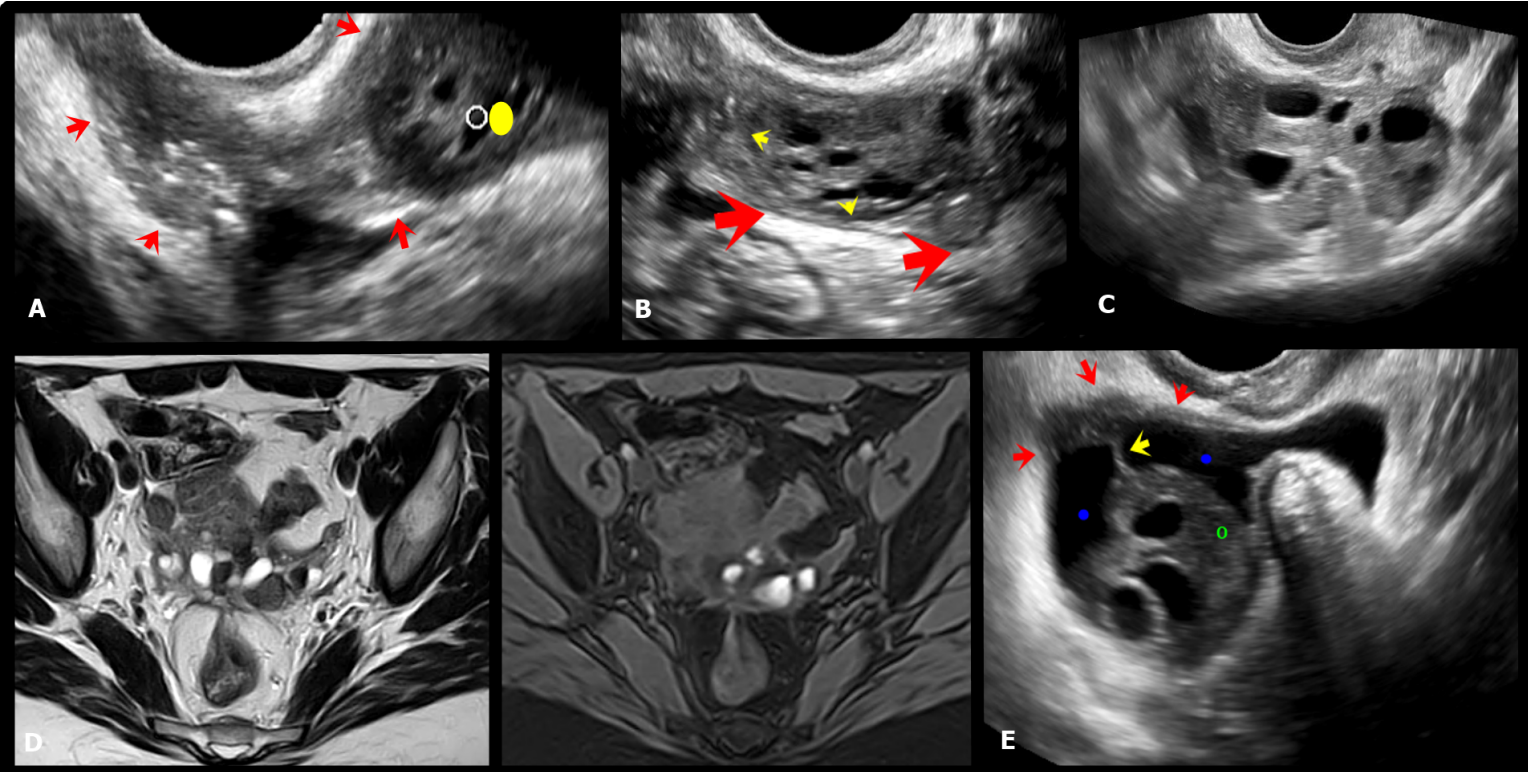
A. Axial oblique transvaginal ultrasound (TVUS) image shows a large hypoechoic endometriotic plaque (red arrow) with irregular and ill-defined margins, infiltrating the peritoneum of the left paracervical region and the ipsilateral ovarian fossa (yellow dot). B. Sagittal oblique TVUS image shows a hypoechoic endometriotic lesion infiltrating the left ovarian fossa (red arrows) and ovarian capsule (yellow arrows). C. Axial TVUS shows “kissing ovaries” as a result of posteriorisation and retraction of the ovaries out of the adnexa/ovarian fossae into the medial pelvis secondary to deep endometriosis (DE). D. Axial T2W and T1W MR images with fat suppression demonstrating kissing ovaries and multiple bilateral ovarian endometriomas and asd well as a DE plaque in the retrocervical space, involving the uterine torus, uterosacral ligaments and the serosa of the anterior wall of the rectum. E. Axial oblique TVUS image demonstrating indirect signs of the adherence process, with paraovarian inclusion cyst (blue dot) presenting thin septum (yellow arrow) and hypoechoic endometriotic lesion infiltrating the peritoneum of the ovarian fossa (red arrows).

**Figure 12:**
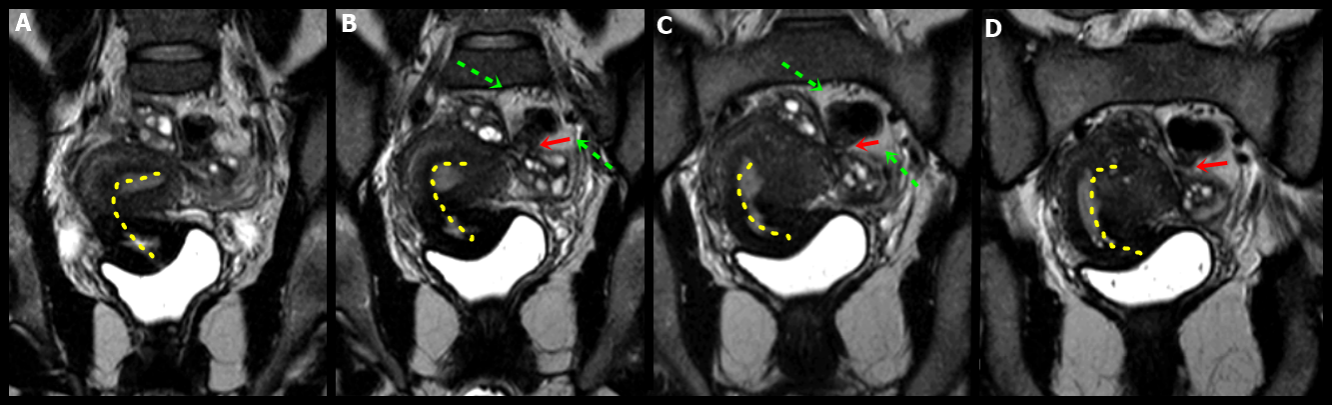
Coronal T2W RM images show a DE in the retrouterine space, causing posteriorisation and medialisation of the ovaries that are located immediately adjacent to each other (red arrows “kissing ovaries”). The implant extends to the left parametrium, adheres and thickens the rectosigmoid colon (green interrupted line). Lateral deviation and retraction of the uterine body and fundus are observed (yellow interrupted line).

The diagnosis is challenging, particularly when endometriomas are absent and endo- metriosis presents exclusively as an adhesive disease. Physicians must know that peritoneal compromise and adhesions are more frequent than we think, even more common than ovarian endometriomas(Redwine, 1999) Hence, it is important to be familiar with the indirect signals of pelvic adhesions (Table IX, Figure 9,10). The “kissing ovaries” sign is seen in cases of adherent bilateral endometriomas, fixed to the posterior face of the uterus (Figures 11, 12).

**Table IX:** Indirect signals of adhesions

Technique	Signals	
	Kissing ovaries (US - MRI)	Bowel loop angulations (US - MRI)
Transvaginal Ultrasound and Magnetic Resonance Imaging	Intraperitoneal inclusion cysts (US - MRI)	Focal augmented thickness of bowel diameter (US - MRI)
	Posterior displacement of uterus (fundal retroversion) and ovaries (US - MRI)	Hydrosalpinx (US - MRI)
	Elevation of posterior vaginal fornix (US - MRI)	Dynamic real time signs (US)
	Loss of fat planes between structures (MRI)	Spiculated low signal intensity strands on T1-W and T2-W (MRI)

**Figure 13:**
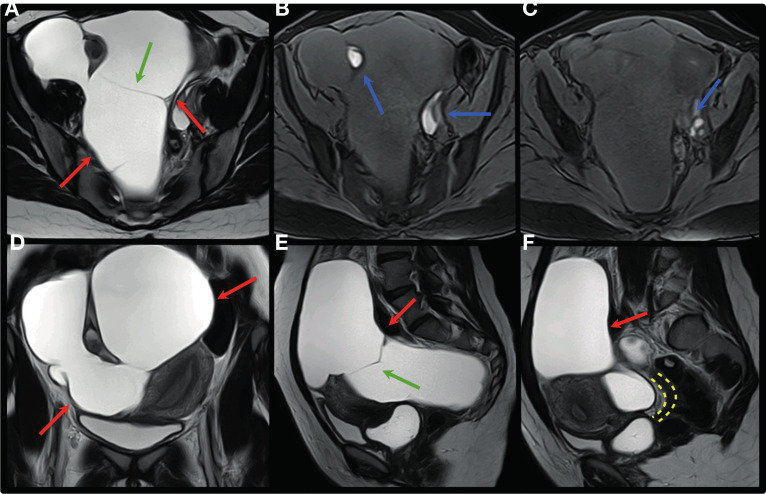
Axial, coronal and sagittal T2W (A, D, E, F) and axial T1W MR images with fat saturation (B,C) show large cyst of peritoneal inclusion occupying practically the entire pelvic cavity (red arrows), with some fine septations (green arrows), bilateral ovarian endometriomas (blue arrows) and deep endometriosis infiltrating the anterior rectosigmoid wall (yellow interrupted line).

**Figure 14:**
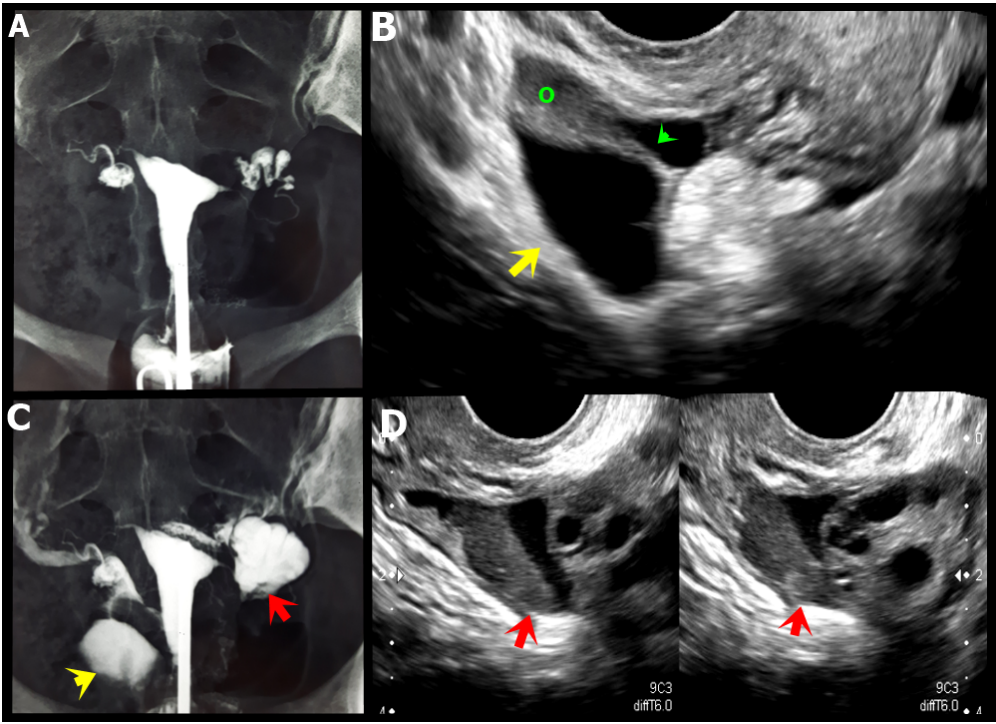
A, C. Hysterosalpingography showing indirect signs of adhesion process in both adnexal regions, characterised by absence of free distribution of the contrast (yellow and red arrows). B. Axial oblique transvaginal ultrasound (TVUS) image demonstrating right para-ovarian peritoneal inclusion cyst with fine septum (green arrow).D. Axial oblique TVUS image shows a hypoechoic endometriotic lesion infiltrating the left ovarian fossa (red arrows) associated and loculated fluid in this location.

The peri-ovarian intraperitoneal inclusion cysts consist of pelvic fluid retention be-tween ovarian peritoneum, uterus and/or posterior cul-de-sac (Figure 13,14).

The presence of a centrally entrapped ovary with peri-ovarian adhesions and inclusion cysts has been called “the spider in a web” sign and it is highly suggestive of peritoneal adhesions (Redwine, 1999). The dynamic real time signs are represented by two main findings:

1. Absent “sliding sign” with the TVUS probe. Pressure is applied against the cervix to see how the anterior rectum slide freely over the posterior aspect of the cervix and posterior vaginal wall.
2. Adherent uterus and/or ovaries to surrounding structures.

## Standardisation of deep endometriosis preoperative evaluation

Standardisation of an operative procedure (surgery or imaging) is an essential component due to their inherent benefits for quality and safety. It has been demonstrated that this process helps to eliminate errors due to omission and prevent bias, provide benchmarks to determine when corrective actions are required, facilitate training by providing regular steps that can be taught, practiced and evaluated, create a common language to describe a specific process so that it can be understood and communicated between surgical teams and preserve the knowledge in time (Guerriero et al., 2016).

One big concern related to deep endometriosis is the lack of universal standard operative methods for ultrasound terminology, anatomical description, disease extension and report. To overcome this two classification systems have been proposed: IDEA group proposal and the new #Enzian classification.

## International Deep Endometriosis Analysis (IDEA) group proposal: A systematic sonographic assessment of pelvic endometriosis

With the objective of standardising the operative evaluation and reporting of the ultrasound findings in patients with suspected pelvic endometriosis, thirty experienced clinicians, gynaecological, advanced laparoscopic surgeons and radiologists with an interest in the diagnosis and management of pelvic endometriosis, from over 16 countries established criteria for the systematic assessment of endometriosis involving the female pelvic structures, and published their consensus (Guerriero et al., 2016). By performing a comprehensive evaluation of current evidence, this group proposed a “four step” analysis for all women with suspected or confirmed endometriosis with the aim to confirm or exclude the different forms of the disease. The main recommendations of this committee are presented in Table X.

**Table X:** Recommendations of the IDEA consensus for assessment of endometriosis.

Area	Factors	Recommendations
Sonographic steps	Evaluation of uterus and adnexae	Uterus: normal, reduced or fixed. Adenomyosis features must be searched and described by using the MUSA proposal. Endometriomas: Measure in three orthogonal planes - Follow IOTA description - Kissing ovaries
	Soft markers	Evaluation of specific-site tenderness, fixed ovaries, sactosalpinx Their presence is suggestive of superficial endometriosis and adhesions
	Status of the Pouch of Douglas	Use of the real-time ultrasound-based on "Sliding sign" A negative sign (absence of smooth glide between retrocervix - anterior rectal wall) is considered as obliteration of the pouch of Douglas.
	Search for DE nodules in Compartments	Anterior: Transducer in anterior fornix (Bladder - uterovesical region - ureters) Posterior: Transducer in posterior fornix (USLs, recto-vaginal septum, Recto-vaginal nodules, posterior vaginal fornix, anterior rectum, sigmoid)
Anterior Compartment	Bladder	Scan with small amount of urine (to reduce false-negatives) Analyze 4 zones: Trigone, base, dome and the extra-abdominal bladder. DE : Hypoechoic linear or spherical lesions with or without regular contours involving muscularis
	Uterovesical Region	Absence of sliding sign ( Anterior fornix/Uterus): Obliteration (+) Sign of adhesions, not necessary endometriosis
	Ureters	Evaluate in the sagittal plane, from the urethra towards the pelvic Sidewall Endometriosis stricture: Dilated long tubular hypoechoic structures. Measure of distance between distal ureteric orifice and stricture zone. Always scan the ipsilateral kidney.
Posterior compartment	DE Nodule	Hypoechoic thickening of bowel/vagina wall Hypoechoic solid nodules, variable in size and contour regularity
	USL	Place probe in posterior fornix in the midline sagittal plane and then sweep inferolateral to the cervix Measure in three orthogonal planes
	Rectovaginal septum	DE in rectovaginal space below the line passing along the lower border of the posterior lip of the cervix Usually and extension of a DE nodule from posterior vaginal wall, anterior rectal wall or both Measure in three orthogonal planes - Distance to anal verge should be measured
	Rectovaginal nodules	Hourglass-shaped or “Diabolo-like”: DE encompassing the posterior vaginal fornix and the anterior rectal wall
	Posterior vaginal fornix	Thickening or discrete nodule found in the hypoechoic layer of the vaginal wall (forniceal endometriosis) Measure in three orthogonal planes
	Anterior rectum	Bowel DE: Thickening of the hypoechoic muscular propria or hypoechoic nodules. Morphological description: 4 types ( Regular, comet sign, moose antler sign, comet and moose, pulling sleeve sign)
	Rectosigmoid junction	Anatomical location: Lower- anterior rectal, Upper-anterior rectal, Rectosigmoidal junction, Anterior sigmoid.
	Sigmoid	Measure in three orthogonal planes - Distance to anal verge should be measured- Mushroom cap sign: Retraction within the rectosigmoid DE lesion (understimation of real length)
	Pouch of Douglas obliteration	Complete or partial : Bilateral / unilateral negative sliding sign Anatomical location: Retrocervical, Mid-posterior, Fundus, Mid-anterior, lower anterior
	Doppler evaluation	No prospective data about its role in DE. Recommended as an adjunct in bowel DE (differential diagnosis with cancer)
Others	Sonovaginography (Saline or gel)	Create an acoustic window- Better visualisation of vaginal walls and anterior/ posterior vaginal fornices 60-120 mL saline solution injected using a Foley catheter - 20-50 mL ultrasound gel (without bubbles) using a 20 mL syringe
	Transrectal sonography	Only when transvaginal ultrasound is impossible or inappropriate
	Tridimensional sonography	Insufficient data-Promising results

The main conclusions of the group included:

1. Trans-vaginal ultrasound is the first line tool for DE and pouch of Douglas obliteration.
2. Success of sliding sign exploration and identification of rectal DE nodules is directly proportional to the operator’s experience.
3. This nomenclature will allow an adequate standardisation of image diagnostic studies, in this way we would be able to compare results worldwide.

## The new ENZIAN classification

The Enzian classification is the result of a consensus process and is based on the opinion of a panel of known gynaecologists and sonographers with extensive expertise in the diagnosis and therapy of endometriosis.

The Enzian classification was initially proposed as a classification for DE using three compartments:

A - vagina, rectovaginal space (RVS)
B - uterosacral ligaments (USL)/cardinal ligaments/pelvic sidewall
C - rectum
F - describes far locations such as the urinary bladder (FB), the ureters (FU), and other extragenital lesions (FO).

The new #Enzian classification additionally covers the involvement of the peritoneum (P), ovary (O), other intestinal locations (sigmoid colon, small bowel; FI), as well as adhesions, involving the tubo-ovarian unit (T) and, optionally, tubal patency (Keckstein et al., 2021).

It is the first classification to universally describe superficial and deep endometriosis, ovarian endometriosis and adenomyosis by using a classification system that can be applied by gynaecologists, surgeons, sonographers, and radiologists following the same principles.

## Discussion

Transvaginal ultrasound has the advantage of offering a dynamic assessment of pelvic anatomy, evaluating the mobility of the pelvic organs. Therefore, it is possible to sus-pect the presence of pelvic adhesions in almost all anatomical compartments. Moreover, Moro et al. (2019) described how ultrasound plays an important role in the surveillance of premenopausal and postmenopausal patients as well as pregnant women with endometriosis, leading to the best management in these subgroups of patients. Ten- derness during transvaginal ultrasound examination can give us additional valuable information about the disease. However, as discomfort and pain can affect up to 25% of patients examined (Schiffmann et al., 2014), it is clearly a disadvantage when compared to MRI examination.

Kelly et al. (2020) showed that, when comparing ultrasound and MRI learning curves for diagnosis of DE, ultrasound trainees had positive learning curves in more anatomical locations (bladder, adenomyosis, overall bowel DE, frozen pelvis) than the radiology/MRI trainees (bladder, adenomyosis). This may indicate that when assessing bowel DE, the learning curve for ultrasound is faster compared to the learning curve for MRI.

Endometriomas can appear in a wide range of imaging findings on TVUS, from the classic “ground glass or snow storm” pattern, to the atypical presentation with variable degrees of intracystic bleeding, hyperechogenic intracystic areas, liquid levels, thickened or thinner walls and irregular contours (Feldstein et al., 1999).

Teixeira et al. (2013) evaluated indirect ultrasound signals of ovarian endometriomas in 50 patients with histological confirmation. The most frequent findings were the “classical features” of endometrioma in 85% of patients, ovarian adhesions in 59% and periadnexal fluid collections in 29%. Just fifteen percent had a hyperechogenic cyst wall foci on the exploration.

Following all those endometrioma signals, Van Holsbeke et al. (2010) found that the correct diagnosis is made with a sensitivity and specificity between 62–73% and 94–98%, respectively. Meanwhile, subjective diagnosis by an experienced radiologist showed equal specificity, with higher sensitivity (88.5%), probably due the use of other clinical information .

Adhesions are frequently associated with endometriosis, and their presence is important for an adequate preoperative assessment. In addition, they are a major factor in the incomplete surgery and final reoperation rate (Nezhat et al., 1995; RCOG Guidelines, 2006). Peritoneal adhesions have been directly related to infertility, ectopic pregnancy and chronic pelvic pain. It has been described that the fixation of an “irregular ovarian shape” to the uterus or other pelvic organs would be signs of adhesions directly related to the endometriotic process (Teixeira et al., 2013). To obtain these signs, the procedure consists in trying to demonstrate a reduction of ovarian mobility and the impossibility of separation from surrounding structures using the sliding sign. When this finding appears on the posterior surface of the uterus, it usually indicates obliteration of posterior cul-de-sac. Furthermore, when the “kissing ovaries” sign appears, severe adhesion syndrome and high intestinal involvement is likely to be present (Ghezzi et al., 2005).

Exacoustos et al. (2003) correlated the TVUS findings to predict stage III and IV endometriosis (including pelvic adhesions), and found sensitivity and specificity of 86%/82% for stage III and 76%/91% for stage IV.

Using a negative sliding sign on TVUS, Guerreiro et al. (2010) reported a 90% sensitivity and specificity for the final presence of uterine and ovarian fixation in the intraoperative time. Similarly, Reid et al. (2013) described a 83% sensitivity and 97% specificity for the diagnosis of posterior cul-de-sac obliteration. The diameter of the endometrioma as a predictor of pelvic adhesions remains controversial (Guerriero et al., 2010).

Meanwhile, MRI can provide direct signs of adhesions (peritoneal band or “sheet-like” structures) or indirect signals including the focal hypo-intense lesions of pre-peritoneal fat line, linear or curve-linear soft tissue strands between organs or peritoneal surfaces, and focal clusters of fluid (Ghonge and Ghong, 2014).

In a study of 57 women with 1.5T MRI, Kataoka et al. (2005) reported a sensitivity and specificity of 77% and 50% respectively for pelvic adhesion syndrome, and 68% and 76% for cul-de-sac obliteration. Later, Manganaro et al. (2012) using the 3.0T MRI described a higher sensitivity (93%) and specificity (75%) for the same disease location. Lienemann et al. (2000) using functional cine- MRI in 27 patients reported a 87% sensitivity and 92% specificity for diagnosing intra-abdominal adhesions.

For intestinal involvement, both TVUS and MRI has shown similar sensitivity and speci-ficity diagnosing rectum lesions. Bazot et al. (2004) reported a global MRI sensitivity and specificity of 84% and 99% respectively.

For ultrasound, Di Giovanni et al. (2018) published a prospective series of 328 patients with bowel DE nodules preoperatively studied with both transvaginal and abdominal ultrasound, and found a 100% sensitivity and 91% specificity.

The major advantages of MRI at this location is the diagnosis of the multifocal and higher involvement, over the rectosigmoid junction, situations where the TVUS has shown lower diagnostic sensibility. This is an important issue, because it is known that multifocal and right intestinal endometriotic implants can present in up to 55% and 28% respectively (Piketty et al., 2009).

A newer technique for the evaluation of colorectal involvement is the contrast enhanced magnetic resonance colonography, using a tridimensional T1-W images with fat saturation, pre and post gadolinium. Scardapane et al. (2011) found a sensitivity and specificity over 95 % for bowel wall involvement.

Tran-Harding et al. (2018) made an interesting correlation between the imaging findings in noninvasive procedures (TVUS and MRI) and the histologic features showing that patients with endometriosis have an estimated 1–2% chance of developing ovarian cancer. Endometriosis and clear cell and endometrioid subtypes of epithelial ovarian cancer are genuinely linked. The pathogenesis for the malignant transformation of endometriosis is unknown. (Jones et al., 2019).

Finally for urinary system endometriosis, Bazot et al. (2004) reported an 88% MRI sensitivity and 99% specificity for bladder involvement, with a 98% of final diagnostic accuracy.

## Conclusion

Preoperative ultrasound and MRI diagnosis following standard evaluation procedures enhance the odds of a surgical success (complete surgery). It is imperative to do an adequate analysis and interpretation of all indirect signals, including at least the absence of sliding signs, inclusion peritoneal cysts, hyperechogenic ovarian wall foci with associated thickening of the peritoneal layer at the ovarian fossae, a fixed ante- or retroverted uterus, medial or posterior ovarian deviation, bowel retractions or angulations, and elevation of vaginal fornix. Although TVUS is the primary line exploration, both exams are complementary and must be used together when necessary, according to their advantages and disadvantages, for a comprehensive evaluation of pelvic endometriosis. The use of an adequate mapping protocol focused on systematic evaluation and reporting of direct and indirect signs of endometriosis is crucial for any surgical planning, leading to a correct and rationale surgical procedure which increases the benefits of the procedure.
